# Evaluating the performance of the Xpert HPV assay in detecting HPV positive cases in Morocco

**DOI:** 10.1016/j.tvr.2025.200318

**Published:** 2025-04-17

**Authors:** Said Ali Yerim, Youssef Chami Khazraji, Rachid Bekkali, Maria Bennai, Nassiba Bahra, Imane Chaoui, Fatima Zahra Chellat, Zineb Gaizi, Nabil Tachfouti, Anas Benabdellah, Bouchra Belkadi, Mohammed Attaleb, Mohamed Amine Berraho, Mohammed El Mzibri

**Affiliations:** a Biology and Medical Research Unit, Centre National de L’Energie, des Sciences et Techniques Nucléaires (CNESTEN), Rabat, Morocco; b Microbiology and Molecular Biology Laboratory, Faculty of Sciences, Mohammed V University, in Rabat, Morocco; c Fondation Lalla Salma– Prévention et Traitement des Cancers, Rabat, Morocco; d Cancer Research Institute, Fez, Morocco; e Laboratory of Epidemiology and Public Health, Faculty of Medicine, Sidi Mohammed Ben Abdellah University, Fez, Morocco

**Keywords:** PCR, GeneXpert, Sequencing, Screening, Cervical cancer, Human papillomavirus

## Abstract

Recently, the World Health Organization recommended integrating HPV testing into cervical cancer screening programs globally. This study aimed to compare the GeneXpert assay with PCR-sequencing for HPV detection and genotyping to assess the feasibility of incorporating HPV molecular testing into cervical cancer screening. A total of 1000 women aged 30 or 40 from rural and urban areas across four regions in Morocco with high sexually transmitted infection prevalence were recruited. After excluding 21 invalid tests, DNA testing on the remaining 979 samples showed an HPV prevalence of 4.0 % (39/979) by PCR and 5.0 % (49/979) by Xpert, with an overall prevalence of 5.7 % (56/979) when combining both techniques. The concordance rate between the tests was 97.5 %. Notably, the Xpert HPV assay was highly efficient in detecting HPV, with nearly all identified HPVs being high-risk oncogenic types, predominantly HPV16, 18, 31, 35, and 45.

The Xpert HPV assay has demonstrated excellent analytical performance, making it a reliable option for HPV detection in vaginal and cervical swabs. Its integration into primary cervical cancer screening programs could significantly enhance the early detection of HPV-positive cases, thereby strengthening the screening framework and potentially reducing both the incidence and mortality of cervical cancer. Future studies should focus on confirming these results and exploring the utility of this method in conjunction with other diagnostic tools such as visual inspection with acetic acid (VIA) for a comprehensive assessment of its effectiveness in real-world settings.

## 1 Introduction

Cervical cancer is the fourth most common cancer in women globally, with around 660,000 new cases and 350,000 deaths in 2022, and the highest incidence and mortality rates occur in low- and middle-income countries [1]. Human papillomaviruses (HPV) are widely recognized as the etiologic factor for cervical cancer development, and they are also implicated in neoplasms of the head and neck—particularly oropharyngeal squamous cell carcinomas—as well as other malignancies such as breast, bladder, colorectal, prostate, and penile cancers [2]. HPV is a common sexually transmitted infection that nearly all sexually active individuals encounter at some point in their lives, typically without symptoms due to immune-mediated viral clearance. However, persistent infection with high-risk HPV (hrHPV) is the primary factor driving abnormal cell formation, the initiation of precancerous lesions, and progression to cervical cancer [3].

More and more countries are shifting from cervical cytology-based screening to HPV-based screening due to its higher sensitivity. Worldwide, HPV detection and genotyping is widely applied in medical laboratories to support cervical cancer screening—whether in co-testing with cytology, for triage of atypical squamous cells of undetermined significance (ASC-US), or for follow-up after colposcopic evaluation. Notably, the World Health Organization recommends HPV testing for primary cervical cancer screening in low- and middle-income countries [4], as it offers higher sensitivity than cytology or visual inspection with acetic acid for detecting cervical neoplasia [5,6].

During the last few decades, molecular techniques for HPV detection have evolved rapidly, both conceptually and methodologically, leveraging significant scientific advances and technological progress. Numerous molecular approaches and platforms for HPV detection and genotyping have been developed, which are now implemented in cervical cancer screening programs in most developed countries as well as many developing ones. Among these platforms, the Xpert HPV assay (Cepheid, Inc., Sunnyvale, CA) has attracted considerable attention for its simplicity, versatility, and sensitivity. It is a qualitative real-time PCR test targeting an 80–150 bp segment in the E6/E7 region, capable of detecting 14 high-risk HPV types. In the assay, HPV16 is detected independently, HPV18 and HPV45 are pooled, and the remaining high-risk HPV types (31, 33, 35, 39, 51, 52, 56, 58, 59, 66, and 68) are reported collectively [7]. Xpert HPV has undergone extensive evaluation and validation, demonstrating high sensitivity and specificity in identifying underlying high-grade disease, both for clinician- and self-collected samples [[8], [9], [10]].

In Morocco, despite substantial efforts, HPV infection remains a significant public health concern, with cervical cancer ranking second after breast cancer among women, at an incidence rate of 18.6 per 100,000 persons/year and an estimated mortality rate of 13.5 cases per 100,000 persons [11]. To address the high prevalence of cervical precancerous lesions and cancer, the Moroccan Ministry of Health has established an integrated program based on three components: early screening (using visual inspection with acetic acid and Pap/cytology tests), prophylactic vaccination of girls aged 9–12 years, and effective treatment combining radiotherapy and chemotherapy. In line with WHO recommendations [4], Moroccan health authorities are now introducing molecular HPV detection and genotyping as a primary screening approach, aiming to overcome the shortcomings of cytology-based programs. Therefore, this study was designed to evaluate the feasibility of integrating molecular testing into cervical cancer screening and to compare GeneXpert results with PCR and sequencing-based methods for both medically- and self-collected samples from women in rural and urban settings in detecting HPV genotypes for epidemiological perspective.

## 2 Material and methods

### 2.1 Study population

In this prospective study, 1000 women aged 30 or 40 were recruited from rural and urban areas of four Moroccan regions with high rates of sexually transmitted infections (Tangier, Beni Mellal, Inezgane, and Taroudant) [[12], [13], [14], [15]]. Women came to the health center for consultation, screening or any other reason. Recruitment followed specific inclusion and exclusion criteria, with pregnant women, those diagnosed with uterine disease, individuals with a personal history of cervical cancer, and those with debilitating illnesses (tuberculous miliaria, congestive heart disease, renal insufficiency or uncontrolled hypertension/diabetes, impaired cognitive function) excluded. Ethical approval was obtained from the Ethics Committee of Medical Research at the Faculty of Medicine and Pharmacy in Rabat (Ref 31–22), and the study was conducted with the support of the Moroccan 10.13039/100009647Ministry of Health and the Regional Office of the 10.13039/100004423WHO in Morocco. All participants were informed that their involvement was voluntary, that they could withdraw at any time, and that their HPV status would remain confidential. Written informed consent was obtained from each participant.

### 2.2 Study design and sampling

**Fig. 1:**
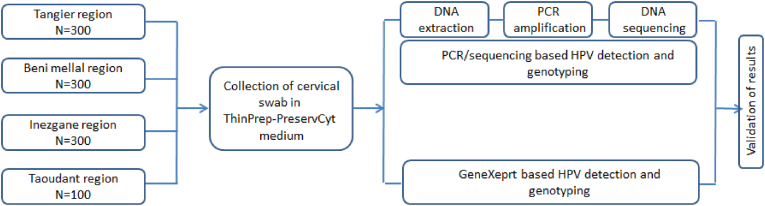
Study design for evaluation of GeneXpert assay performance.

Scraped cervical cells were auto-sampled under or without supervision by qualified nurses or collected by experienced nurses, using cervix brush. Overall, 870 women were sampled by health professionals and only 130 have accepted self-sampling. All women accepting self-sampling have received a short training including the posture to be adopted to ensure adequate sampling of vaginal swabs or single-use brushes by a healthcare professional. After sampling, the brushes are placed in 20 ml of ThinPrep-PreservCyt medium and sent to the Reference Healthcare Centre of the concerned province. Immediately, samples were tested by GeneXpert and the remaining samples were kept at −4 °C for DNA extraction and HPV detection and genotyping by PCR – sequencing approach (Fig. 1).

### 2.3 GeneXpert HPV assay

GeneXpert HPV assay was conducted according to the manufacturer's protocol. From each sample, 1 ml of cervical specimen collected in ThinPrep-PreservCyt medium was subsequently loaded into the sample chamber of the Xpert HPV cartridge and inserted in the GeneXpert®module for HPV detection. Experimental procedure including extraction, amplification and data analysis was performed within 60 min and results are reported in the machine screen as either negative or positive either with HPV16, HPV18-45 and/or other hrHPVs 31, 33, 35, 39, 51, 52, 56, 58, 59, 66 and 68. All positive results were done with their respective Ct values.

### 2.4 PCR and sequencing based HPV detection and genotyping

DNA was extracted from ThinPrep-PreservCyt medium using the standard SDS-proteinase K–phenol–chloroform method [16]. PCR was performed using GP5^+^/6^+^ primers to amplify a 150 bp fragment of L1 gene. PCR amplification was done in a 20 μl containing 15 μM of each primer, 2 μl of genomic DNA and 1X of x-VITA Hot Start 2X Master Mix (Gquene). The amplification mixtures were first denatured at 94 °C for 5 min, following by thirty-five cycles of PCR with denaturation at 94 °C for 35 s, primer annealing for 35 s at 60 °C and primer extension for 35 s at 72 °C. At the end of the last cycle, mixtures were incubated at 72 °C for 7 min. For every reaction a positive control, using HPV confirmed DNA, and a negative control were included.

Positive PCR products were purified by the ExoSaP IT^R^ clean up system (USB, USA). Direct sequencing of amplicons was performed using Big Dye Terminator kit (version 3.1) (Applied Biosystem, Foster City, CA, USA) that includes dideoxynucleotides labeled with four fluorochromes of different colors. For each PCR product, sequencing was performed using the GP5+ primer. To eliminate the excess of labeled ddNTPs, sequencing reaction products were purified by the ethanol-formamide method. Direct sequencing of amplified PCR products was performed on an ABI 3130xL Genetic Analyzer (Applied Biosystems). The resulting chromatograms were manually edited to ensure sequence accuracy using Sequencing Analysis v5.4 software. This sequence was used for nucleotide-nucleotide BLAST analysis (blastn) against known HPV genotype sequences in the GenBank database (www.ncbi.nlm.nih. gov/BLAST/).

### 2.5 Statistical analysis

A descriptive analysis was performed to characterize the different types of HPV infections identified in samples, with a presentation of the frequencies of each genotype. Additionally, an analysis was conducted to assess the analytical performance of the GeneXpert test. Measured performance included relative sensitivity, specificity, positive predictive value (PPV) and negative predictive value (NPV). For each measure, 95 % confidence intervals were calculated to evaluate the precision of the estimates. The analyses were conducted using SPSS software, version 26.

## 3 Results

**Fig. 2:**
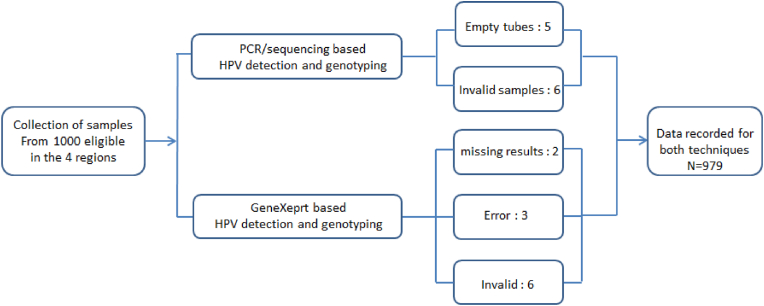
Flow diagram depicting the details of steps involved in the study.

In this comparative study, a total of 1000 cervical samples were collected, and flow diagram showing all details of steps involved in the testing along with the number of samples the number of samples retained for comparison or discarded due to technical problems is reported in Fig. 2.

After quality control, 21 samples were excluded due to technical issues. With the in-house molecular technique, 11 samples were missed due to the absence of cells/insufficient volume in the ThinPrep-PreservCyt medium (5 samples) or the inability to amplify extracted DNA either with HPV primers or with primers targeting the β-globin, used as a housekeeping gene (6 samples). On the GeneXpert workstation, 11 samples failed: 9 samples showed errors (N = 3 errors during sample preparation due to probe control failure and N = 6 invalidated tests during PC) and 2 samples were missed. Of note, 1 sample was not amplified by PCR and has shown an error during sample preparation in the GeneXpert assay. Of the 979 remaining samples, 39 (4.0 %) were tested positive for HPV DNA by the in-house PCR method and 49 (5.0 %) by GeneXpert (Table 1). Using PCR as the reference, GeneXpert showed relative sensitivity of 82.1 %, specificity of 98.2 %, positive predictive value of 65.3 % and negative predictive value of 99.2 % (Table 2).

**Table 1:** Comparison of the positivity rates of GeneXpert assay and HPV DNA amplification.

		GeneXpert results		Total
		Positive	Negative	
PCR results	Positive	32	27	39
	Negative	17	923	940
	Total	49	930	979

**Table 2:** Evaluation of GeneXpert assay according to HPV DNA amplification results.

Parameters	No.	%	95 % CI
Relative sensitivity	32/39	82.1 %	[70.1–94.1]
Relative specificity	923/940	98.2 %	[97.4–99.0]
Relative PPV	32/49	65.3 %	[52.0–78.6]
Relative NVP	923/930	99.2 %	[98.6–99.8]

For the 32 samples that tested positive with both methods, genotyping comparisons revealed a 90.6 % (29/32) concordance rate (Table 3). The most frequently detected high-risk genotypes in these concordant cases were HPV16, HPV18, and HPV31, while HPV33, HPV45, and HPV51 each accounted for a smaller proportion. A few discrepancies were observed, where GeneXpert identified double infections or hrHPV types that PCR-sequencing ultimately confirmed as HPV16 or HPV18.

**Table 3:** Comparison between GeneXpert and DNA sequencing for HPV genotyping.

N°	GeneXeprt Data	Sequencing results	N°	GeneXeprt data	Sequencing results	N°	GeneXeprt data	Sequencing results
1	Other HrHPV	51	12	Other hrHPV	31	23	18–45	18
2	18–45	18	13	Other hrHPV	31	24	18–45	18
3	Other hrHPV	52	14	18–45	45	25	18–45	45
4	Other hrHPV	31	15	Other hrHPV	68	26	16	16
5	Other hrHPV	31	16	Other hrHPV	31	27	Other hrHPV	31
6	18–45	18	17	18–45	18	28	16	16
7	Other hrHPV	56	18	Other hrHPV	33	29	16	16
8	18–45	18	19	Other hrHPV	31	30	16 and Other hrHPV	16
9	18–45	18	20	Other hrHPV	31			
10	Other hrHPV	16	21	16	16	31	Other hrHPV	51
11	16	16	22	Other hrHPV	33	32	Other hrHPV	18

Among the seven samples negative by GeneXpert but positive by PCR, sequencing identified HPV16, HPV31, HPV35, and HPV67, with one undetermined hrHPV. Conversely, for samples negative by PCR but positive by GeneXpert, HPV16, HPV18-45, or other undetermined hrHPV types were detected.

## 4 Discussion

In this study, an overall HPV prevalence of 5.7 % (56/979) was observed using both GeneXpert and PCR-sequencing. Nearly all detected HPV types were high-risk genotypes (5.6 %), with HPV16, 18, 31, 35, and 45 predominating, alongside the less common HPV67. These findings align with regional reports from Tunisia (7.3 %) [17], Algeria (6.3 %) [18] and Egypt (11.5 %) [19] and they reflect previously documented variations in HPV prevalence across different geographic areas [20,21].

Morocco has worked to combat cervical cancer through a comprehensive program encompassing vaccination, early detection, and effective treatment. Recently, the Ministry of Health has considered introducing GeneXpert for molecular HPV detection and genotyping, a critical step in strengthening the existing screening infrastructure in line with WHO recommendations. Although HPV detection with PCR and genotyping by Sanger sequencing is widely employed in epidemiological research to identify circulating genotypes [[22], [23], [24]], this is the first study to compare these standard approaches directly with GeneXpert for HPV detection. By evaluating GeneXpert's performance, we aim to determine its added value for primary cervical cancer screening in Morocco. It should be noted that this study is not intended to validate or evaluate the clinical performance of the Xpert HPV test, which has already been done in previous studies.

Our results indicate that the overall HPV prevalence and the rate of high-risk HPV have significantly declined in Morocco, suggesting a positive impact of the national cervical cancer control program in curbing the spread of HPV among sexually active individuals. This decrease in HPV infection parallels a reduction in reported cervical cancer cases, as national registries (Greater Casablanca and Rabat) document notable declines in cervical cancer incidence from 2004 to 2017 [[25], [26], [27], [28], [29], [30], [31], [32]]. The incidence of cervical cancer standardized to the world/national population reported by the Rabat registry in 2005 was 15/12.3 per 100,000, and has dropped to 12.7/12.1 during the 2009–2012 period [25,27]. Similarly, in the Greater Casablanca Register—one of the largest in North Africa covering both urban and rural populations—incidence fell from 15/12.7 and 16.3/14.1 (during 2005–2007 and 2008–2012) to 10.2/9.2 (2013–2017), and then to 8.8/8.4 (2018–2021). During these periods, cervical cancer's share among all cancers declined from 12.8 % to 11.2 %–8.1 % and 6.5 %, placing it third after breast and thyroid cancer [[29], [30], [31], [32]]. This success reflects an integrated approach involving multiple stakeholders and supporting organizations, aiming to expand screening among sexually active women and ensure that most women with precancerous or cancerous lesions receive the appropriate treatment.

The Xpert HPV assay demonstrated excellent analytical performance in detecting high-risk HPV genotypes, achieving 97.5 % concordance with the PCR/sequencing approach. Its relative sensitivity and specificity were 82.2 % and 98.2 %, respectively, with superior detection among positive cases. Previous comparative studies have evaluated Xpert against clinically validated FDA-approved tests such as HC2, Cobas 4800, and Aptima, generally reporting good to excellent concordance [33]. For instance, Rabaan et al. showed an almost perfect overall concordance (98.5 %) between Xpert and HC2, with corresponding high-risk HPV prevalence rates of 7.9 % and 9.3 % [34]. Another evaluation comparing Xpert, Cobas 4800, and Aptima on 982 vaginal (self-collected) and cervical samples recorded a total concordance and an overall 11.6 % high-risk HPV prevalence [8]. In that study, both Xpert and Cobas showed similar high levels of sensitivity and specificity, while Aptima exhibited slightly higher specificity at the expense of lower sensitivity [8].

Other studies have examined the performance of the Xpert HPV assay in detecting CIN2+ lesions among women referred for colposcopy, reporting sensitivity, specificity, PPV and NPV values of 90.4 %, 43.1 %, 29.4 %, and 94.5 %, respectively, comparable to Cobas (90.4 %, 39 %, 28 %, and 94 %) and HC2 (81.6 %, 46.5 %, 28.5 %, and 90.6 %) [7]. These findings highlight the strong analytical performance of Xpert HPV and its potential to replace other assays for routine HPV detection and genotyping.

Despite these favorable results, our study uncovered some discrepancies. Specifically, 1.7 % (17/979) of samples were Xpert-positive but PCR-negative, while 0.7 % (7/979) were PCR-positive but Xpert-negative. In two cases identified by Xpert as harboring "other hrHPV," DNA sequencing detected HPV16 and HPV18 instead. Similar inconsistencies have been noted elsewhere [35,36]. These variations may stem from technical differences: Xpert conducts automatic lysis and purification within its cartridge, ensuring high-quality extract for PCR, whereas our in-house method relies on manual DNA extraction. Furthermore, Xpert targets the E6/E7 region of the viral genome, whereas the PCR-based approach targets the L1 region. Additionally, the case involving HPV67 could not be identified by Xpert since that genotype, deemed less oncogenic, is not included in its detection panel.

Of particular interest, only 0.9 % (9/1000) of tests failed on the GeneXpert platform, a rate that is relatively low compared to those reported in other studies, which range from 1.9 % (22/1161) to 24.0 % (36/150) [37,38], This suggests that tests were executed correctly and reliably.

Most validated commercial tests currently available require sequential testing based on their platform designs, which poses risks for test invalidation or failure, potentially delaying result delivery and negatively impacting the screening program. In contrast, the Xpert HPV platform offers a turnaround time of 60 min, with tests that can be run independently and concurrently. Additionally, the closed-cartridge design of Xpert HPV reagents significantly reduces human intervention, thereby minimizing the risk of contamination and false positives [33,[39], [40], [41]]. Of particular interest, many studies have reported the compatibility of Xpert HPV test with the self-testing. Moreover, the portability of the GeneXpert system is of potential advantage in facilitating large screening, particularly during awareness campaigns in very remote locations [42,43].

This study provides valuable insights into the performance of the Xpert HPV test for screening and genotyping HPV in cervical specimens, underscoring the benefits of integrating this effective and easy-to-use technique into cervical cancer control programs as a primary screening tool for detecting high-risk HPV. The technique does not require complex infrastructure and can even be operated by non-laboratory-trained personnel, facilitating its widespread adoption throughout the country. However, the study has some limitation mainly the absence of histopathological results, which would enable exploration of the associations between HPV genotypes and cytological status, and the very few HPV positive samples obtained among the analyzed cases. Moreover, it is difficult to obtain strong evidence about sensitivity with this low amount of HPV positive samples. Also, using Sanger sequencing to characterize HPV genotypes does not differentiate between single and co-infections, as this method mainly identifies the dominant genotype within a sample. Additionally, it lacks an economic evaluation to assess the cost-effectiveness of implementing Xpert HPV testing within the program, considering the relatively high costs of the tests.

## 5 Conclusion

The Xpert HPV test has demonstrated high reliability and strong concordance with PCR-sequencing in detecting HPV in vaginal and cervical swabs, making it highly suitable for integration into primary cervical cancer screening programs. As an FDA-approved method, its excellent analytical performance not only aids in the accurate detection of HPV-positive cases but also enhances the existing screening framework, contributing to further reductions in both the incidence and mortality of cervical cancer. Future studies should focus on evaluating the effectiveness of point-of-care Xpert HPV testing in conjunction with visual inspection with acetic acid (VIA) for detecting and treating precancerous cervical lesions, to further substantiate its utility in real-world settings.

## CRediT authorship contribution statement

**Said Ali Yerim:** Writing – original draft, Methodology, Investigation. **Youssef Chami Khazraji:** Writing – review & editing, Validation, Resources, Investigation, Conceptualization. **Rachid Bekkali:** Writing – review & editing, Funding acquisition, Conceptualization. **Maria Bennai:** Writing – review & editing, Funding acquisition, Conceptualization. **Nassiba Bahra:** Formal analysis, Data curation. **Imane Chaoui:** Writing – review & editing, Methodology, Investigation. **Fatima Zahra Chellat:** Formal analysis, Data curation. **Zineb Gaizi:** Formal analysis, Data curation. **Nabil Tachfouti:** Writing – review & editing, Formal analysis. **Anas Benabdellah:** Writing – review & editing, Formal analysis. **Bouchra Belkadi:** Writing – review & editing, Validation, Conceptualization. **Mohammed Attaleb:** Writing – review & editing, Visualization, Investigation. **Mohamed Amine Berraho:** Writing – review & editing, Resources, Investigation, Conceptualization. **Mohammed El Mzibri:** Writing – review & editing, Supervision, Investigation, Funding acquisition, Conceptualization.

## Ethical approval

The study protocol was approved by the Ethics Committee of Medical Research at Faculty of Medicine and Pharmacy in Rabat (Ref 31–22)

## Funding

The study was funded by the Fondation Lalla Salma-Prévention et Traitement des Cancers.

## Declaration of competing interest

The authors declare that they have no known competing financial interests or personal relationships that could have appeared to influence the work reported in this paper.

## Data Availability

No data was used for the research described in the article.
